# A potential role for trace amines in the treatment of septic shock

**DOI:** 10.1016/j.jointm.2025.09.003

**Published:** 2025-12-23

**Authors:** Kenneth J. Broadley, Alexander C. Voisey, William R. Ford, Harrison D. Broadley

**Affiliations:** 1 Division of Pharmacology, School of Pharmacy & Pharmaceutical Sciences, Cardiff University, Cardiff, UK; 2 Royal College of Surgeons of Ireland in Bahrain, Busaiteen, Kingdom of Bahrain

**Keywords:** Septic shock, Vasopressor treatment, Trace amines, Blood vessel, Animal models

## Abstract

Sepsis involves an unregulated response to bacterial infection, the inflammatory response leading to persistent hypotension, leading to a precipitous fall in arterial blood pressure. The major trigger for the hypotension in sepsis and septic shock is the release of lipopolysaccharide (LPS) from the cell wall of gram-negative bacteria, which generates a major inflammatory response. LPS activates toll-like receptors (TLR4), leading to the release of an array of inflammatory mediators, which most notably activate inducible nitric oxide (NO) synthase and release copious amounts of NO. This is responsible for the hypotension. Despite the development of a large number of newer drugs for treating sepsis, none has emerged as superior to existing treatments. Early vasopressor therapy remains an integral life-saving strategy to treat the hypotension. Noradrenaline remains the vasopressor of choice; however, it has a number of limitations which are discussed. Improvements in vasopressor therapies are required and research from the authors is used to advance the case for using trace amines, such as β-phenylethylamine (PEA). Although usually regarded as sympathomimetic amines, PEA and related amines such as amphetamine and ephedrine exert vasoconstrictive effects via trace amine-associated receptors (TAARs), particularly when administered by infusion. The sympathomimetic and TAAR vasoconstrictor actions are demonstrated on anesthetized rat blood pressure. Unlike noradrenaline, PEA is not a universal vasoconstrictor; it also dilates other vessels, including mesenteric vascular beds. This would provide a superior profile of activity for use in restoring blood pressure in sepsis. The ability of PEA to reverse the vasodilator action of LPS is demonstrated in a simple *in vitro* blood vessel model. This review therefore opens the possibility of using trace amines for restoring blood pressure and organ perfusion in septic shock.

## Introduction

Sepsis is defined as life-threatening organ dysfunction arising from an unregulated host response to bacterial infection.^[^^1^^]^ The whole-body inflammatory response leads to persistent hypotension, which in septic shock leads to a fall in mean arterial blood pressure to <60 mmHg. The development of hypotension prevents tissue oxygenation demands from being adequately met.^[^^2^^]^ The major trigger for hypotension in sepsis and septic shock is the release of lipopolysaccharide (LPS), a cell wall component of gram-negative bacteria associated with an inflammatory response.^[^^3^^]^ Circulating LPS interacts with toll-like receptors (TLR4) found on most human cells, and its stimulation leads to downstream activation of an array of enzymes and release of vasoactive mediators (Table 1). Activation of cyclooxygenase also leads to the release of prostaglandins (PG) PGE_2_ and PGD_2_ and leukotrienes (LT) LTB_4_ and LTC_4_. These activate inducible nitric oxide synthase (iNOS),^[^^4^^]^ resulting in rapid production of large amounts of nitric oxide (NO), which causes relaxation of vascular smooth muscle.^[^^5^^]^ The powerful vasodilatation by NO accounts for the dramatic life-threatening falls in blood pressure.^[^^6^^]^

**Table 1:** Vasoactive and inflammatory mediators released by lipopolysaccharide.

Mediator	Vascular response	Receptor	Antagonists	Release from
Nitric oxide	dilates all	TLR4	L-NAME (NOS inhibition)	vascular wall
Thromboxane A_2_ (B_2_ inactive product)	Vasoconstrictor, platelet aggregation down-regulates iNOS	TP	Seratrodast, AA2414, terutroban	Platelets, monocytes
Leukotriene C_4_ (cysteinyl leukotriene)	Vasodilator vasoconstrictor- renal, gastric, coronary	LT_1_	zafirlukast (Accolate)	Macrophages, mast cell, basophil, eosinophil
Leukotriene B_4_	vasodilator (e.g., skin)	BLT	CP105,696, LY255283	
Prostaglandin E_2_	vasodilator, renin release	EP_2_	AH6809, SC19220, AH23848	Macrophages
Prostaglandin D_2_	Vasodilator - coronary vasoconstriction	DP_2_ or EP_2_	Laropiprant	Macrophages, mast cell
Cytokines (TNF-α. IL8, IL6, IL1, IFN-γ, IL1β, IL6, IL8)	Pro-inflammatory	Type 1 cytokine receptor, TNF-receptor	Infliximab (TNF-α)	Macrophages
Bradykinin	Endothelium-dependent vasodilator	B_2_	MEN16132, icatibant	Gut wall, mast cell, kidney
5-HT	Vasoconstriction/vasodilatation	5HT_2A_	Ketanserin	Platelets
Endothelin	vasoconstrictor	ET_A_/ET_B_	Bosentan	Endothelium

5-HT: 5-Hydroxytryptamine; B_2_: Bradykinin; BLT: Leukotriene B receptor; DP_2_: Prostaglandin D_2_ receptor; EP_2:_ Prostaglandin E2 receptor; ET_A_/ET_B_: endothelin receptors; iNOS: Inducible nitric oxide synthase; l-NAME: N_ω_-nitro-l-arginine methyl ester; IFN: Interferon; IL: Interleukin; LT_1_: Leukotriene; NOD: Nitric oxide synthase; TLR: Toll-like receptor; TNF: Tumor necrosis factor; TP: Thromboxane;

### Current Management of Hypotension in Sepsis with Vasopressors

The treatment of sepsis and septic shock involves a multitarget approach including antibiotics, fluid resuscitation, β-adrenoceptor antagonists, and anti-inflammatory agents. However, hypotension and cardiac dysfunction are the main life-threatening features of sepsis and septic shock,^[^^7^^]^ and early vasopressor therapy remains an integral life-saving strategy. Noradrenaline is the vasopressor of choice, but mortality rates remain high with its use.^[^^1^^]^ A further issue is that noradrenaline is a universal vasoconstrictor, in that it constricts all vascular beds, including the mesentery. This results in impaired tissue perfusion of vital abdominal organs, leading to organ damage.^[^^8^^]^ Another limitation of vasoconstrictors is that they are less effective in sepsis because sepsis causes hyporeactivity of blood vessels to vasoconstriction.^[^^9^^]^ α_1_-adrenoceptors become downregulated in sepsis and, therefore much higher doses of α_1_-adrenoceptor agonists, such as noradrenaline, are required to raise blood pressure. These high doses may induce atrial fibrillation and may alter sepsis-associated changes in the immune response.^[^^10^^]^ Thus, there remains a substantial potential for novel improved therapies for the management of hypotension in septic shock, and one such group that we have identified is the trace amines.

### Trace amines

Sympathomimetic amines such as β-phenylethylamine (PEA), tyramine, amphetamine, and ephedrine (Table 2) are so-called because they mimic the effects of sympathetic nerve stimulation.^[^^11^^]^ They exert their pharmacological effects by releasing noradrenaline from neuronal vesicular storage sites of sympathetic nerve endings and have therefore been classified as indirectly acting sympathomimetic amines.^[^^12^^]^ This distinguishes them from directly acting sympathomimetic amines such as noradrenaline, adrenaline, and phenylephrine (Table 2), which act directly on adrenoceptors. In the cardiovascular system, indirectly acting amines increase heart rate by releasing stored noradrenaline onto cardiac β-adrenoceptors. They also cause increases in blood pressure through released noradrenaline, causing a predominant vasoconstriction in cutaneous and mesenteric arterioles via stimulation of α-adrenoceptors.

**Table 2:** Agents interacting with trace-amine receptors.

Sympathomimetic amines	Receptor
Directly acting on adrenoceptors	
Noradrenaline	α/β
Adrenaline	α/β
Phenylephrine	α
Indirectly acting sympathomimetic amines^⁎^	
Tyramine	TAAR1
PEA	TAAR1
(dex)Amphetamine	TAAR1
Methamphetamine	Not known
MDMA	TAAR1
Phenylpropanolamine	Not known
Cathinone	TAAR1
Octopamine	TAAR1
Mixed direct/indirect	
Ephedrine	Not known
Tryptamine	TAAR1

^⁎^ Release of stored noradrenaline.

MDMA: 3,4-Methylenedioxymethamphetamine; PEA: β-Phenylethylamine; TAAR: Trace amine-associated receptors.

Oral administration to humans of tyramine,^[^^13^^]^ ephedrine, ^[^^14^^]^ cathinone^[^^15^^]^ and the synthetic amines, phenylpro-panolamine,^[^^16^^]^ amphetamine,^[^^17^^]^ methylphenidate,^[^^18^^]^ and 3,4-methylenedioxymethamphetamine (MDMA) (“ecstasy”)^[^^19^^]^ causes increases in blood pressure. In animals, intravenously administered tyramine is well known to cause increases in blood pressure in rats^[^[20], [21], [22]^]^, cats,^[^^12^^,^^20^^]^ dogs,^[^^23^^]^ and rabbits.^[^^24^^]^ Other amines, such as phenylpropanolamine,^[^^25^^]^ MDMA (“ecstasy”),^[^^26^^]^ ephedrine,^[^^21^^]^ methamphetamine,^[^^27^^]^ amphetamine, and β-PEA^[^^20^^]^ all cause pressor effects in conscious, anesthetized, or pithed rats. Tyramine, amphetamine, and cathinone cause pressor responses in dogs.^[^^28^^]^ Pressor responses of anesthetized rats to ephedrine^[^^21^^]^ and MDMA^[^^26^^]^ were partially antagonized by phentolamine and prazosin, respectively, and are therefore attributed partially to α-adrenoceptor stimulation.

**Figure 1:**
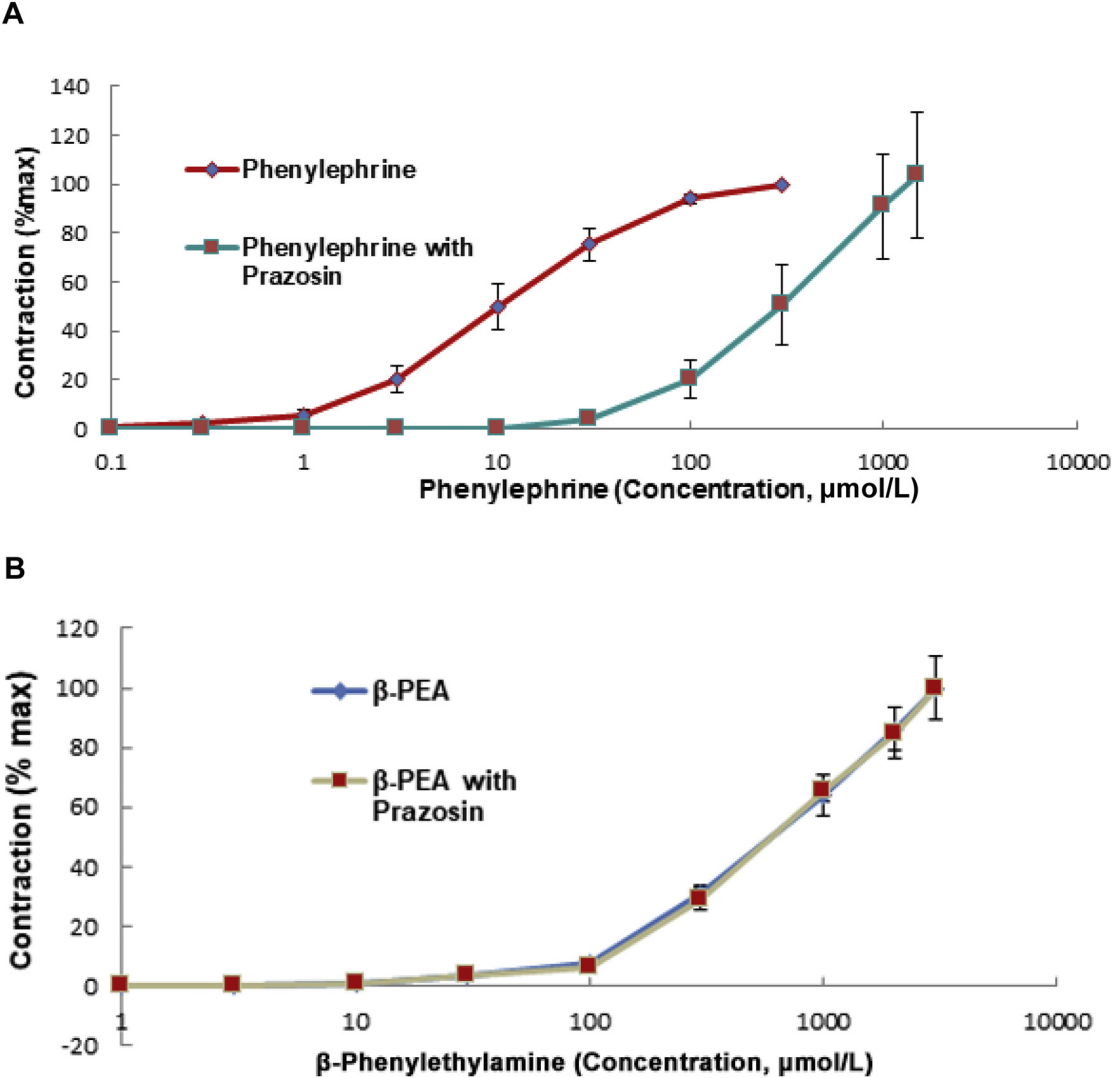
Cumulative concentration-response curves were constructed for phenylephrine and sequential curves for β-PEA in the absence (diamond) and repeated in the presence (squares) of prazosin. Effect of prazosin on the contractions of guinea-pig aortic rings to phenylephrine and β-phenylethylamine. A: Prazosin (1µmol/L) blocks the contractile responses of guinea-pig aortic ring to the α-adrenoceptor agonist, phenylephrine, which are therefore due to stimulation of α-adrenoceptors. B: Prazosin does not block the constrictor responses to β-phenylethylamine (β-PEA), which are therefore not due to α-adrenoceptor stimulation. Responses are the mean (±SEM; *n*=4) contractions expressed as a percentage of the contraction to the maximum of the 1st curve. Mean -log EC50 values (±SEM) (pD2) before and after prazosin were compared by paired Student’s t-test. A significant difference (P <0.05) was seen between the pD2 values for phenylephrine but no significant difference for β-PEA. β-PEA, β-Phenylethylamine; EC_50__:_Effective concentration for 50% response; SEM: Standard error of the mean.

More recent evidence suggests that indirect sympathomimetic activity may not entirely explain the cardiovascular effects of these amines.^[^^29^^]^ The vasoconstrictor responses to PEA of rat^[^^30^^,^^31^^]^ and guinea-pig^[^^32^^,^^33^^]^ isolated aorta, pig coronary arteries,^[^^34^^]^ and human saphenous vein and mammary artery^[^^35^^]^ are not inhibited by the α_1_-adrenoceptor antagonist prazosin. Vasoconstrictor responses of coronary artery to tyramine^[^^36^^]^ and to cathinone^[^^37^^]^ are also not inhibited by prazosin. This is illustrated in (Figure 1), where the concentration–response curve for the contractions of guinea-pig aortic rings to the directly acting α-adrenoceptor agonist phenylephrine was shifted to the right by prazosin, indicating that the vasoconstriction was mediated via α_1_-adrenoceptors. The concentration–response curve for contractions to PEA, however, was unaffected and not therefore due to α-adrenoceptor stimulation. The vasoconstriction by PEA has therefore been attributed to a separate group of non-adrenergic receptors known as trace amine-associated receptors (TAARs).^[^^38^^]^ Nineteen individual TAARs for rat, nine TAARs for human, and 16 for mouse have been identified.^[^^39^^]^ However, only TAAR1 and TAAR4 are functionally active. TAAR1 is the only human variant activated by classical amines, including PEA, tyramine, and tryptamine.^[^^38^^,^^40^^]^ TAAR4 responds to these but is not functional in humans. TAAR1 receptor protein has been identified by Western blotting and TAAR1 receptor mRNA by reverse transcription polymerise chain reaction (RT-PCR) in rat aorta.^[^^31^^]^ TAAR4 is weakly expressed in rat aorta.^[^^31^^]^ TAAR1 is the only subtype expressed in humans and therefore the only target for therapeutic interventions. It is widely expressed in the brain, spinal cord, pancreatic β-cells, stomach, and intestines of humans.^[^^41^^]^ Other studies show low-level localization of mRNA for the human TAAR1 in the brain, moderate levels in the stomach, kidney, and lung, and lower levels in the liver, prostate, skeletal muscle, and spleen.^[^^38^^]^ In rat heart tissue, transcripts for at least five TAAR subtypes, including TAAR1, have been detected by RT-PCR.^[^^42^^]^ Blood vessels other than rat aorta do not appear to have been examined for expression of TAARs.

**Figure 2:**
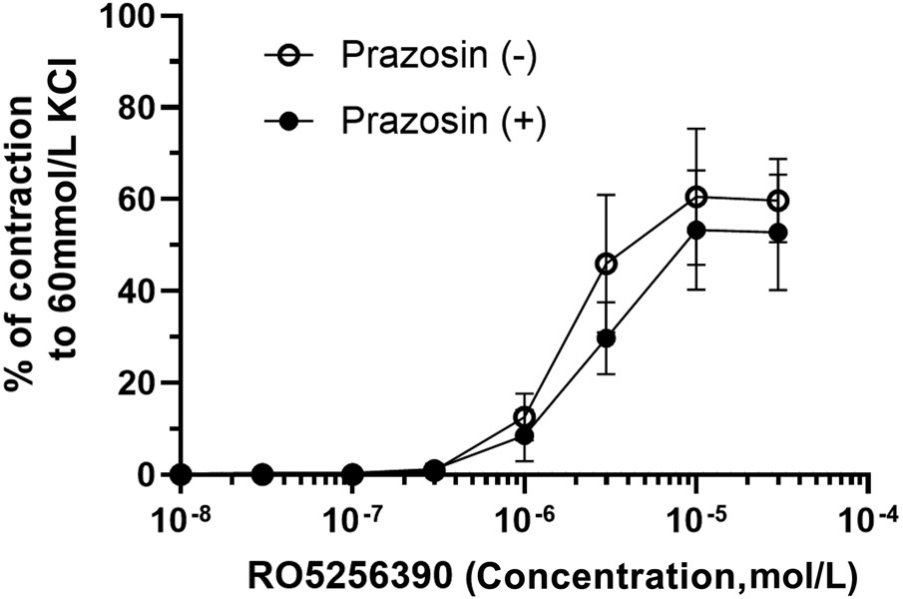
Cumulative concentration-response curves were constructed in the absence (control, ○) or presence of the α_1_-adrenoceptor antagonist prazosin (1 µmol/L, ●). Contractions of rat isolated aortic rings to the selective TAAR1 agonist RO5256390. The selective TAAR1 agonist, RO5256390, causes contractions of rat isolated endothelium-denuded aortic rings, which are NOT blocked by the α_1_-adrenoceptor antagonist, prazosin. The contractions are no therefore due to α_1_-adrenoceptor stimulation. Prazosin had no significant effect on the EC_50_ for RO5256390 (4.4 µmol/L, 0.5–8.3 µmol/L, *n*=5) compared with the control in the presence of DMSO (0.02%) (3.7 µmol/L, 1.1–6.3 µmol/L, *n*=5). Increases in tension are reported as the mean percentage (±SEM) of the maximum contraction to KCl (60 mmol/L). EC_50__:_ Effective concentration for 50% response; DMSO: Dimethyl sulphoxide; SEM: Standard error of the mean; TAAR1: Trace amine-associated receptor 1.

Only a limited number of agents selective for TAARs have been identified or synthesized, and currently, there are only two commercially available agonists or antagonists selective for TAARs.N-(3-Ethoxy-phenyl)-4-pyrrolidin-1-yl-3-trifluoromethyl-benzamide (EPPTB) is a potent selective antagonist for mouse TAAR1.^[^^43^^]^ It is much less potent and less selective for rat and human TAAR1. RO5256390 is a potent selective agonist for TAAR1 with no activity at TAAR4.^[^^44^^]^ In rat aortic rings, RO5256390 produces concentration-related vasoconstriction.^[^^45^^]^ This is not blocked by prazosin (Figure 2) and therefore is not mediated through α_1_-adrenoceptors, presumably acting via TAAR1.

**Figure 3:**
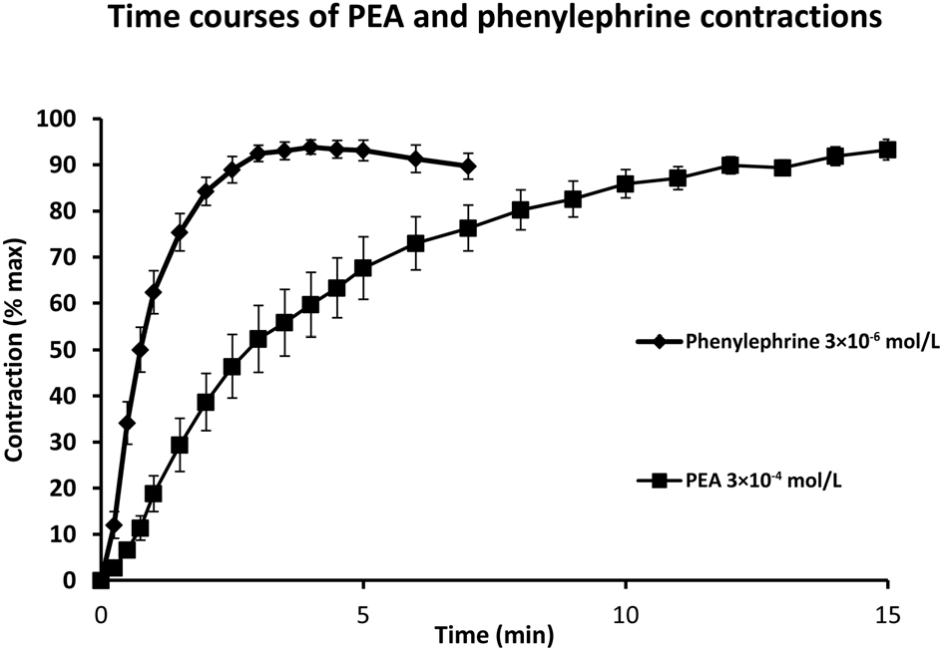
Time courses for the contractions of guinea-pig aortic rings to phenylephrine and PEA. Vasoconstriction of guinea-pig aorta to PEA (■) was slow in onset and delayed in reaching its peak. Vasoconstriction to the α-adrenoceptor agonist, phenylephrine (♦), was more rapid in onset and reached its peak sooner. Mean time courses for the contractions of endothelium-intact guinea-pig aortic rings to submaximal doses of phenylephrine (3 × 10^−^^6^ mol/L, *n*=13) and PEA (3 × 10^−4^ mol/L, *n*=14). These were the 55.5%±6.6% KCl and 40.0%±6.8% KCl of maximum responses, respectively. Responses were obtained in cumulative concentration-response curves and expressed as a percentage of the contraction to KCl (60 mmol/L) added at the maximum. Increases in tension from the maximum contraction to the previous dose are expressed as a percentage of the maximum contraction to that concentration of agonist and the mean (±SEM) plotted against time. PEA: β-phenylethylamine; SEM: Standard error of the mean.

Further evidence that the vasoconstrictions by PEA and phenylephrine are mediated differently comes from the rates of onset of their vasoconstrictor responses in isolated aorta. Vasoconstriction to the α_1_-adrenoceptor agonist, phenylephrine, is rapid in onset but wanes while the amine is still present. The response to 3 × 10^−6^ mol/L of phenylephrine in guinea-pig aorta peaked at (3.9±0.4) min. In contrast, the equivalent vasoconstriction to PEA was slow in onset, sustained, and peaked significantly later at (13.6±1.3) min (Figure 3).

**Figure 4:**
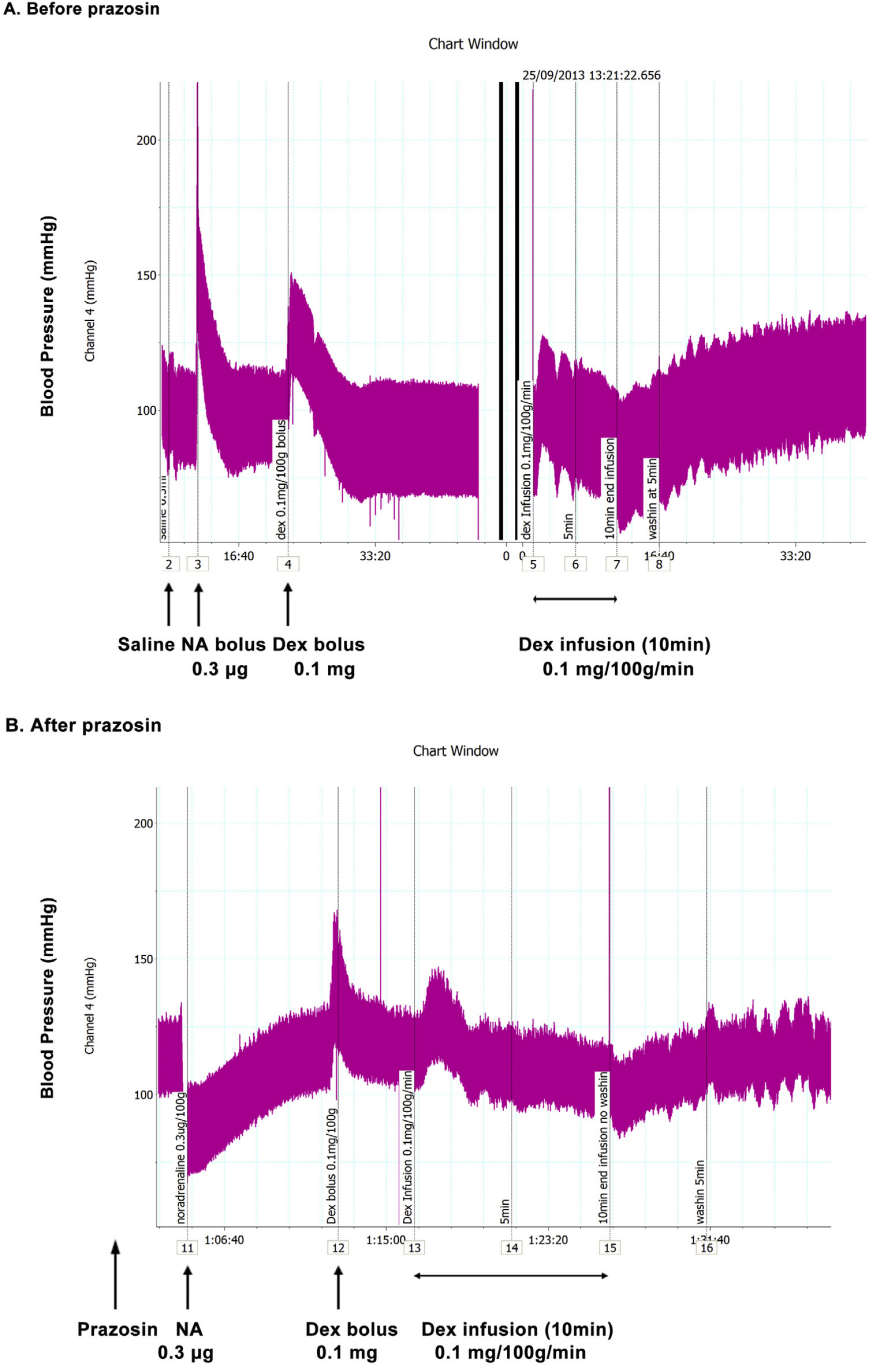
Blood pressure responses of anesthetized rat to dexamphetamine. The α-adrenoceptor antagonist, prazosin, reverses the pressor response to a bolus dose of noradrenaline of anesthetized rat to a fall in blood pressure while reducing that to dexamphetamine. A delayed increase in blood pressure following infusion of dexamphetamine was not blocked by prazosin indicating an α-adrenoceptor-independent pressor response. Dex (0.1 mg/100 g) and infusions (0.1 mg/(100 g·min) for 10 min) were administered to a male Sprague-Dawley rat (320 g) anesthetized with pentobarbitone sodium (60 mg/kg intraperitoneal injection). NA bolus doses (0.3 µg/100 g) were also administered. Arterial blood pressure responses were recorded (A) before and (B) after intravenous administration of the α1-adrenoceptor antagonist, prazosin (0.1 mg/100 g slowly). Dex: Dexamphetamine bolus doses; NA: Noradrenaline.

*In vivo*, the vasoconstriction by PEA and tyramine increases blood pressure after intravenous administration in rats^[^[20], [21], [22]^,^^46^^]^, cats^[^^12^^,^^20^^]^, dogs,^[^[21], [22], [23], [28], [46]^]^ and rabbits.^[^^24^^]^ Blood pressure also increases in humans after administration of tyramine^[^[13], [47]^]^ and phenylpropanolamine.^[^^16^^]^ Most of the evidence indicates that the *in vivo* increases in blood pressure by tyramine, β-PEA, and related amines are due to their indirect sympathomimetic mechanisms. For example, the pressor actions of tyramine are blocked by depletion of noradrenaline stores with reserpine.^[^^12^^]^ In contrast, *in vitro* observations in isolated blood vessels suggest the involvement of TAAR receptors. A possible reason for this discrepancy is that most *in vitro* studies involve immersed tissues, allowing prolonged contact with the amines. In contrast, *in vivo* clinical studies^[^[13], [48], [49]^]^ have usually examined bolus dosing in which there is only a brief peak concentration of agonist in the circulation. Since the rate of onset of TAAR-mediated vasoconstriction by PEA is significantly slower than for the α-adrenoceptor agonist, phenylephrine (Figure 3),^[^^33^^]^ bolus dosing of trace amines *in vivo* may prevent observation of the delayed and slower TAAR1-mediated increases in blood pressure. This observation has been borne out in studies on anesthetized rat blood pressure, where infusions of PEA^[^^46^^]^ and dexamphetamine (Figure 4) produce multiphasic blood pressure responses. An initial rapid-onset increase in blood pressure was short-lived but followed by a slow-onset increase. After stopping the infusion, there was a further persistent increase in blood pressure to both PEA and dexamphetamine (Figure 4). The initial pressor response was antagonized by the α_1_-adrenoceptor antagonist, prazosin, indicating that it is mediated via α_1_-adrenoceptors. In contrast, the delayed and sustained increases in blood pressure by PEA and dexamphetamine were unaffected, indicating that the delayed pressor responses were most likely due to vasoconstriction through stimulation of vascular TAAR1 receptors.

### Potential for Trace Amines in Septic Shock

As described above, one of the main life-threatening complications of sepsis and septic shock is the precipitous fall in arterial blood pressure and consequential underperfusion of vital organs. Vasopressors are the mainstay for reversing arterial hypotension, and noradrenaline is the vasopressor of choice. Phenylephrine and ephedrine have been used by bolus dosing in septic shock,^[^^50^^]^ and phenylephrine has been used in anaphylactic shock.^[^^51^^]^ There is limited information on infusions of these amines; however, phenylephrine infusions have been used to maintain blood pressure during spinal anesthesia for cesarean section.^[^^52^^]^ There is no information on the use of PEA in septic shock.

As pointed out above, noradrenaline has its limitations – it is a universal vasoconstrictor in all vascular beds, including the mesentery, and secondly there is down-regulation of α_1_-adrenoceptors in sepsis so that higher doses of noradrenaline are required. The trace amine PEA, however, does not cause vasoconstriction in the mesenteric vascular bed. In rat isolated perfused mesenteric beds^[^[53], [54]^]^ or endothelium-denuded rat third-order mesenteric arteries^[^^45^^]^ preconstricted with phenylephrine, only a vasodilatation was observed to PEA and tyramine. This vasodilatation was abolished in the perfused mesenteric beds by the nitric oxide synthase (NOS) inhibitor N_ω_-nitro-l-arginine methyl ester (L-NAME) indicating that it was mediated via the release of NO, probably from the vascular endothelium.^[^^54^^]^ Trace amines appear to exert vasodilator responses in most blood vessels to some extent, but in vessels where vasoconstriction dominates, the vasodilatation can only be seen indirectly or when the tone in the vessel is raised by preconstriction. For example, in guinea-pig aortic rings, an underlying vasodilatation is evident by a potentiation of the vasoconstriction to these amines when NO is inhibited with l-NAME^[^^32^^]^ and of their pressor responses in conscious rabbits.^[^^24^^]^ Thus, if PEA were used to raise blood pressure in septic shock, it would not be expected to impair blood flow to vital abdominal organs. Further work is required to establish the relative vasoconstrictor and vasodilator activities of PEA on other vascular beds.

### Pilot Studies with Trace Amines

**Figure 5:**
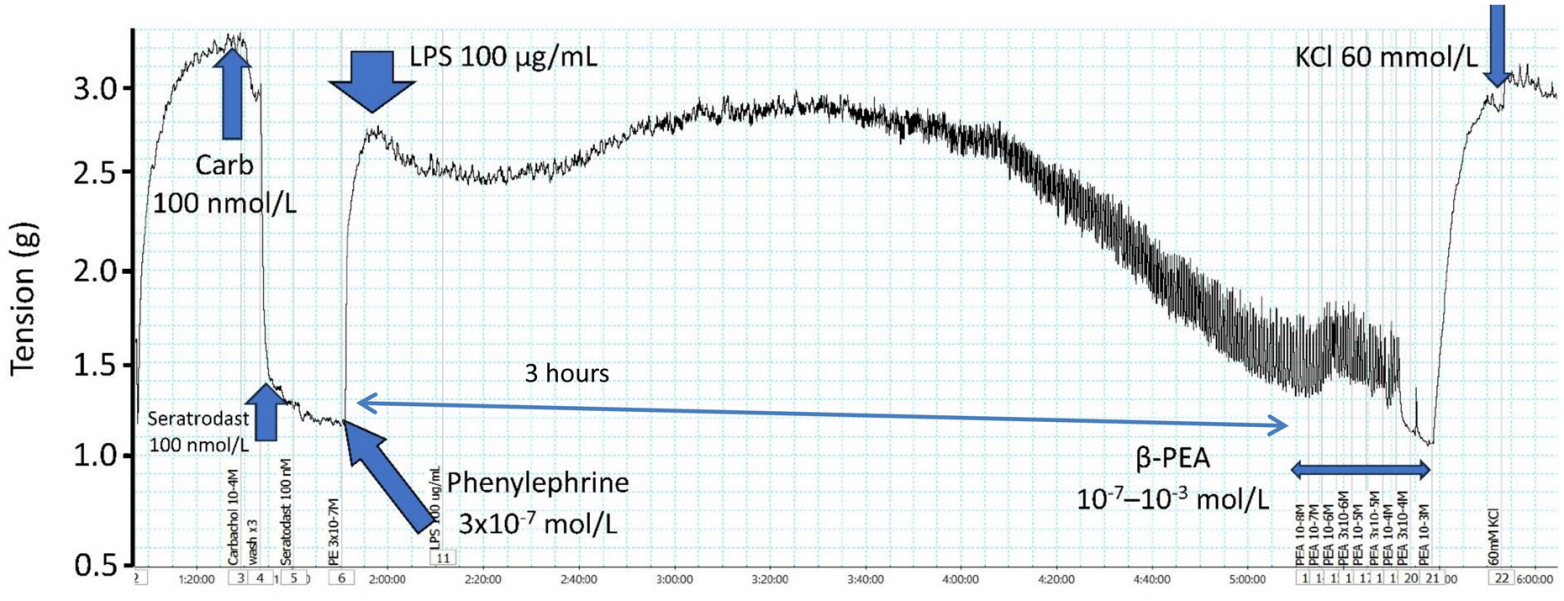
Record of rat aortic ring tension showing preconstriction to phenylephrine and then addition of LPS (100 µg/mL) for 3 h which causes initial further contraction and then vasodilatation back to baseline. Addition of β-PEA in half log increments in concentration (10^−7^–10^−3^ mol/L) at this point caused contraction and restoration of tone. Experiment performed in the presence of thromboxane A_2_ antagonist, seratrodast (100 nmol/L). KCl 60 mmol/L was added at the beginning and end to show the maximum tissue contraction, and Carb (100 nmol/L) added to show a relaxation due to endothelial presence and release of nitric oxide. LPS: Lipopolysaccharide; β-PEA: β-phenylethylamine; Carb: Carbachol.

**Figure 6:**
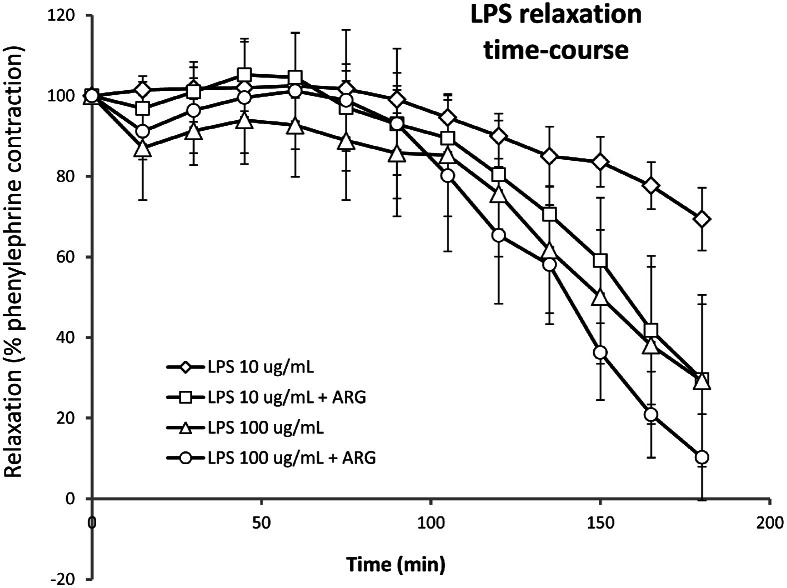
Lipopolysaccharide relaxation of rat aorta. Time course for the relaxation by LPS (10 or 100 µg/mL) of rat isolated aorta preconstricted with phenylephrine (300 µmol/L). The relaxation was enhanced by the ARG (10 µmol/L), indicating that nitric oxide was the predominant mediator of the vasodilatation. Responses were recorded either in the absence or presence of l-arginine. Mean (±SEM; *n*=4) relaxation responses are measured as the change in tension from the peak of the phenylephrine contraction and expressed as a percentage where the peak phenylephrine response is 100%. LPS: Lipopolysaccharide; ARG: Nitric oxide donor l-arginine hydrochloride; LPS: Lipopolysaccharide; SEM: Standard error of the mean.

**Figure 7:**
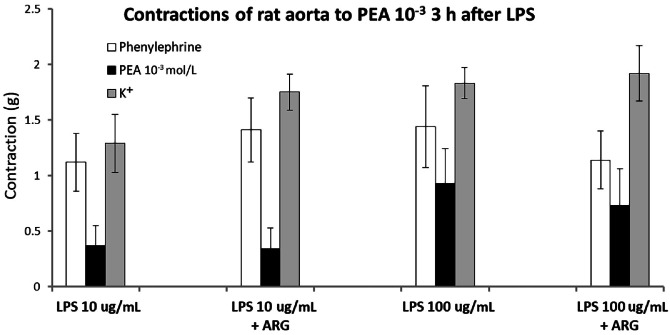
PEA, phenylephrine and K^+^ contractions 3 h after lipopolysaccharide relaxation of rat aorta. PEA restores the contractions of rat isolated aorta that have been reduced to baseline by incubation with LPS. Contractions of rat aortic rings to PEA (10^−3^ mol/L), phenylephrine (3 × 10^−7^ mol/L) and K^+^ (60 mmol/L) were obtained at the end of 3 h exposure to LPS (10 or 100 µg/ml), in the absence or presence of the ARG (10 µmol/L). Aortic rings were preconstricted with phenylephrine (300 µmol/L) before adding the LPS. Mean contractions (±SEM) are measured in g tension. ARG: Aitric oxide donor l-arginine hydrochloride; LPS: Lipopolysaccharide; PEA: β-phenylethylamine;SEM: standard error of the mean.

To evaluate the prospective utility of PEA, other trace amines, or selective TAAR1 agonists in restoring blood pressure and organ perfusion in septic shock, their effectiveness in a model of sepsis needs to be established. It was necessary to mimic the fall in blood pressure caused by LPS, the causative agent released by bacterial infection in sepsis pathology. Because our initial studies have demonstrated the non-adrenergic vasoconstrictor actions of PEA in rat isolated aorta, it was initially necessary to establish a simple *in vitro* model of LPS-induced vasodilatation also in rat isolated aorta. However, there did not appear to be any relevant studies in the literature. Previous studies have pretreated rats with LPS before removing the aorta.^[^[55], [56]^]^ Isolated aortic, tail artery, or mesenteric rings have also been incubated with LPS, and this has been for long periods of 18–24 h^[^[57], [58], [59]^]^ or shorter times (4 h).^[^^60^^]^ The incubation concentration of LPS has ranged from 1 µg/mL to 100 µg/mL. In no studies could we find any measurements of arterial contraction/relaxation during the exposure to LPS. To determine the ability of a vasoconstrictor to reverse LPS-induced vasodilatation *in vitro*, it was necessary to mimic the fall in blood pressure caused by LPS. Therefore, we preconstricted isolated aortic rings with phenylephrine (3 × 10^−7^ ^mol/L^) and then added LPS (10 or 100 µg/mL) to incubate for 3 h (Figure 5). There was an initial increase in tension; the most likely vasoconstrictors released by LPS are thromboxane A_2_, 5-hydroxytryptamine (5-HT), and endothelin (Table 1). Of these, 5-HT is released from platelets and is therefore an unlikely candidate. Thromboxane was the most likely, and experiments were therefore conducted in the presence of the TxA_2_ antagonist, seratrodast. This did not completely inhibit the vasoconstriction, which may therefore have been due to endothelin or residual thromboxane. After about 1 h of incubation with LPS, there was a gradual relaxation that reached baseline after about 3 h. This relaxation could be primarily attributed to NO as it was enhanced by incubation with the NO donor, l-arginine (10 µmol/L) (Figure 6). This was most marked with the lower concentration of LPS (10 µg/mL). After 3 h of incubation with LPS and l-arginine, PEA was added in half-log increments from 10^−7^ mol/L to 10^−3^mol/L. The tension increased and finally reached the original phenylephrine-induced contraction (Figure 7). These pilot *in vitro* studies therefore demonstrated that the trace amine PEA could cause vasoconstriction in isolated blood vessels relaxed by incubation with LPS and thereby restore vascular tone. This observation mimics the desired action of PEA in restoring blood pressure reduced by LPS release in sepsis.

## Conclusions

Sepsis is one of the leading causes of death worldwide. Despite advances in its management and extensive research in the development of new drugs, treatment strategies, and better use of existing drugs, the annual number of cases has continued to rise, and mortality remains stubbornly high. Treatments can be grouped into three approaches: (1) immediate treatment of the causative infection with antibiotics; (2) life-saving reversal of the potentially fatal fall in blood pressure due to the release of bacterial LPS and resultant organ failure through lack of perfusion; and (3) treatment of the inflammatory response. Recent developments in new therapies have focussed on the inflammatory response, but the success rate for this approach has been modest. The available drugs for restoring blood pressure and organ perfusion do not appear to have increased markedly over the past decade, and while the vasopressor drug of choice, noradrenaline, has remained in use for decades, it still has limitations, and there is a need for a more ideal replacement. This review has put forward a case for trace amines, exemplified by PEA. Trace amines such as PEA are vasoconstrictors in most vascular beds, exerting their effect as agonists for TAARs, in the case of vasoconstriction, TAAR1. Unlike most other vasopressors available for septic shock management, PEA is not a universal vasoconstrictor but has vasodilator activity in the mesenteric vasculature and therefore would not be expected to impair perfusion of vital mesenteric organs. Pilot studies presented in this review show for the first time a slow-onset vasodilatation of rat isolated aorta by LPS, which reaches its maximum relaxation after 3 h. At this point, the addition of PEA caused a restoration of tension. Further studies are ongoing to establish that this restoration of vascular tone extends to LPS-induced falls in blood pressure *in vivo,* thus confirming the potential for trace amines in septic shock management.

## CRediT authorship contribution statement

**Kenneth J. Broadley:** Writing – review & editing, Writing – original draft, Supervision, Investigation, Funding acquisition, Formal analysis, Data curation, Conceptualization. **Alexander C. Voisey:** Writing – review & editing, Data curation. **William R. Ford:** Supervision, Funding acquisition. **Harrison D. Broadley:** Writing – review & editing, Formal analysis, Data curation.
